# Low grade fibromyxoid sarcoma: a case report and review of the literature

**DOI:** 10.1186/1749-799X-5-49

**Published:** 2010-07-29

**Authors:** Christina Arnaoutoglou, Marios G Lykissas, Ioannis D Gelalis, Anna Batistatou, Anna Goussia, Michalis Doukas, Theodoros A Xenakis

**Affiliations:** 1 Department of Orthopaedic Surgery, University of Ioannina School of Medicine, Ioannina, Greece; 2 Department of Pathology, University of Ioannina School of Medicine, Ioannina, Greece

## Abstract

Low grade fibromyxoid sarcoma (LGFMS) is a distinctive variant of fibrosarcoma with a high metastasizing potential and sometimes long interval between tumour presentation and metastasis. We present the case of a 50-year-old male who developed a large mass in the posterior aspect of his lower left thigh. The tumor was excised with preservation of the neurovascular structures surrounded by the mass. The tumour measured 11 × 10 × 9 cm and on pathology evaluation was diagnosed as LGFMS. Due to the relative rarity of LGFMS, there is no dedicated protocol regarding follow-up recommendations. In order to early diagnose possible metastasis it is important to inform the patients about the longstanding metastatic potential of the disease.

## Background

Low grade fibromyxoid sarcoma (LGFMS) is a distinctive variant of fibrosarcoma. According to Evans [1], who first described this pathologic entity, LGFMS presents a rare soft-tissue tumour with a high metastasizing potential, despite the benign histologic appearance. The sometimes long interval between tumour presentation and metastasis poses problems for the pathologists, radiologists, and surgeons, with longstanding follow-up being the fundamental principle for tumour management.

Although it is a well-described entity it is speculated that many cases are not diagnosed as LGFMS, so it is still difficult to estimate its exact incidence. These tumours usually occur in the proximal extremities and trunk [2]. Sporadically they may be found in unusual locations, such as the retroperitoneum, head or the chest wall [2,3]. The majority of LGFMS occur in a subfascial location, but in rare occasions the subcutis or dermis may be affected [4]. LGFMS typically involves young or middle-aged adults, but a large number of paediatric cases has also been described [3,5-9].

Herein, we present the case of a 50-year-old male who developed a large LGFMS on the posterior aspect of his lower thigh. Imaging depiction, surgical approach of the mass, and current follow-up recommendations are discussed.

## Case presentation

A 50-year-old male presented with a large tumour on the posterior aspect of his lower left thigh. The mass was growing slowly during the past 3 years, but the patient did not seek for medical treatment until his presentation in our department. Physical examination revealed a firm mobile mass without tenderness, redness or warmth.

**Figure 1:**
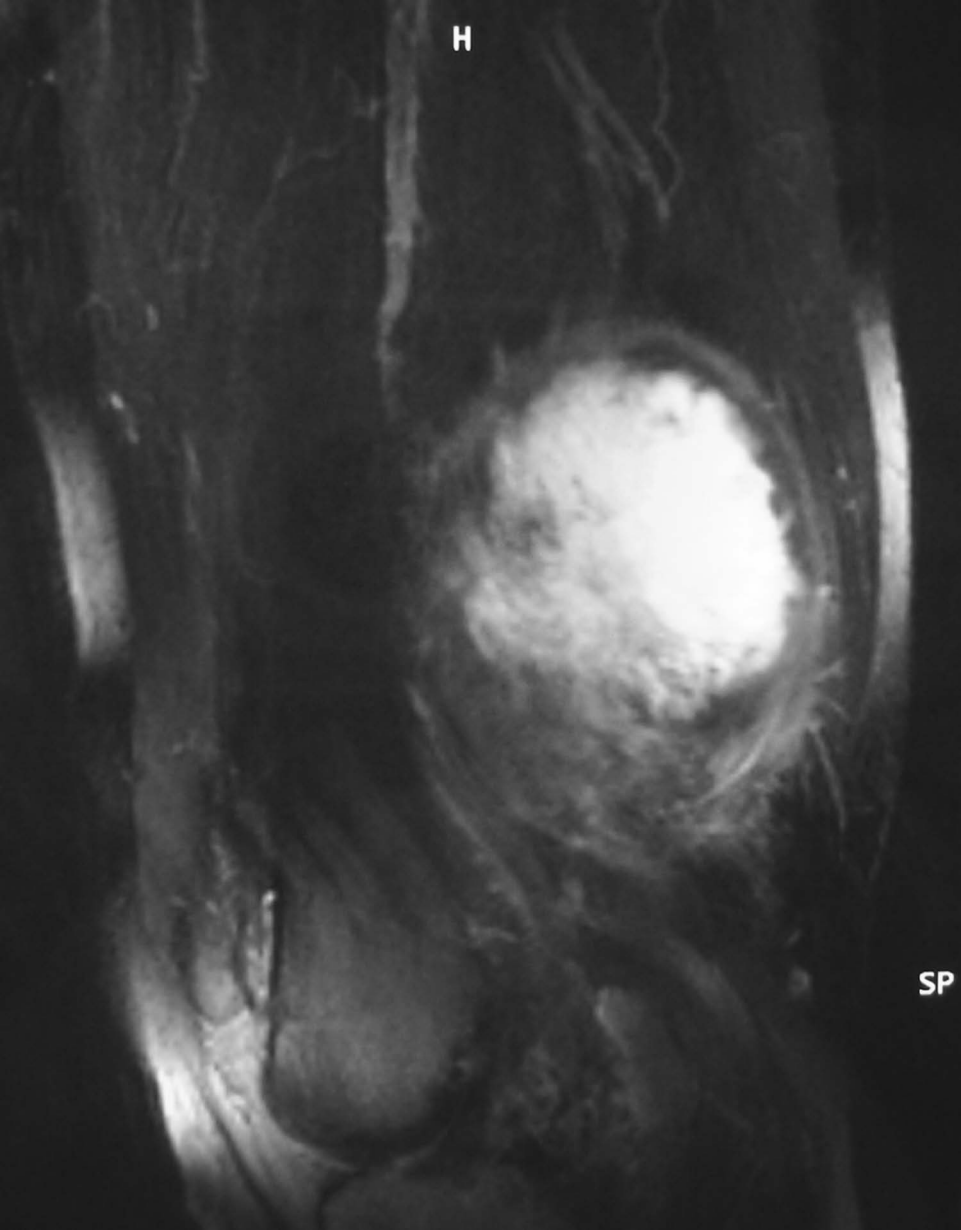
**"T1-weighted, fat-suppressed, contrast-enhanced on a sagittal plane MR image revealed a hyperintense central part and a hypointense irregular wall corresponding to myxoid and fibrous pattern, respectively**."

Laboratory evaluation and plain radiographs were unremarkable. A computed tomography (CT) scan with intravenous administration of contrast material showed a large, heterogenous, low-density mass (Hounsfield units measuring between 15 and 50) surrounding the superficial femoral artery and vein within the Hunter's canal as well as the sciatic nerve at the lower thigh. A mixed density including hypodense and isodense components was evidence on CT. Magnetic resonance imaging (MRI) evaluation of the area also demonstrated a mixed myxoid and fibrous pattern within the tumour. The fibrous component was identified as hypointense areas on T1- and T2-weighted MR images and slightly enhancing on T1-weighted MR images after intravenous administration of gadolinium (Fig. 1). The myxoid component of the mass was recognized as hypointense on T1-weighted MR images and hyperintense on T2-weighted MR images, and avidly enhancing on T1-weighted MR images after gadolinium administration (Fig. 1).

The patient subsequently underwent a wide biopsy for additional information, before surgical resection of the mass. Histological evaluation revealed a moderately cellular lesion consisting of spindle cells with mild atypia and morphological features of fibroblasts, set in a collagenous stroma. The cells exhibited eosinophilic cytoplasm and vesicular nuclei with, the small nucleoli and were arranged in a plexiform or whorled pattern. Mitoses were not found. A very-limited necrotic area was recognized in the periphery of the biopsy specimen. Immunohistochemically, the neoplastic cells were positive for vimentin and focally for SMA. Taking into account the clinical and radiographic findings, malignancy could not be ruled out and a low-grade mesenchymal neoplasm was suggested.

Seven weeks later the tumor was excised using meticulous technique, with preservation of the neurovascular structures surrounded by the mass. No perforation of the vessels or nerves was evident during mass removal. The mass was well-circumscribed, but not encapsuled, making the resection insecure regarding the surgical margins. A drain was placed 1 cm distal of the peripheral end of the surgical incision. Elaborate hemostasis and wound closure was followed. After surgery, the patient experienced no major complications and was discharged 5 days later.

**Figure 2:**
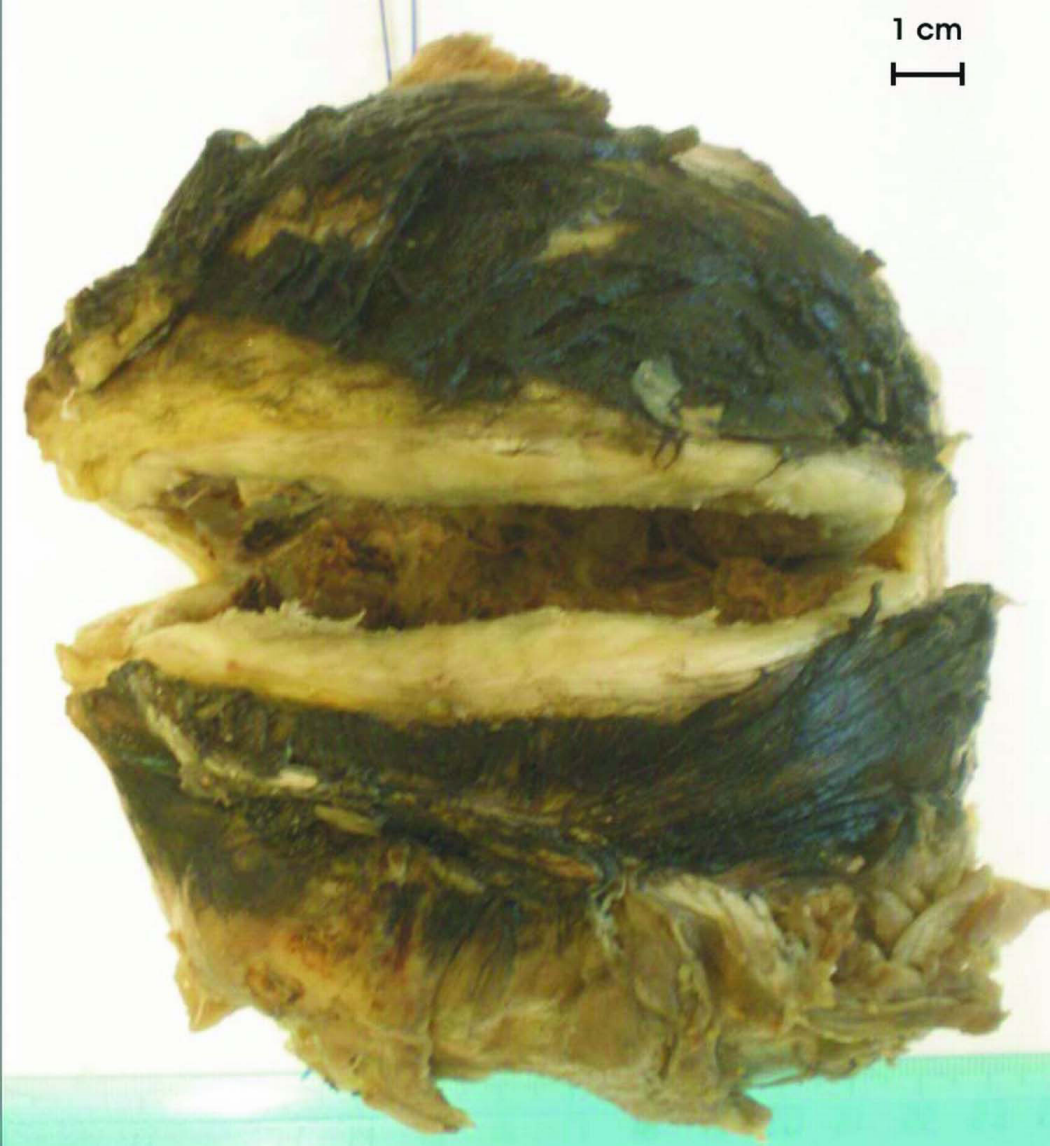
**Gross pathological specimen**. The cut section revealed a cystic formation.

**Figure 3:**
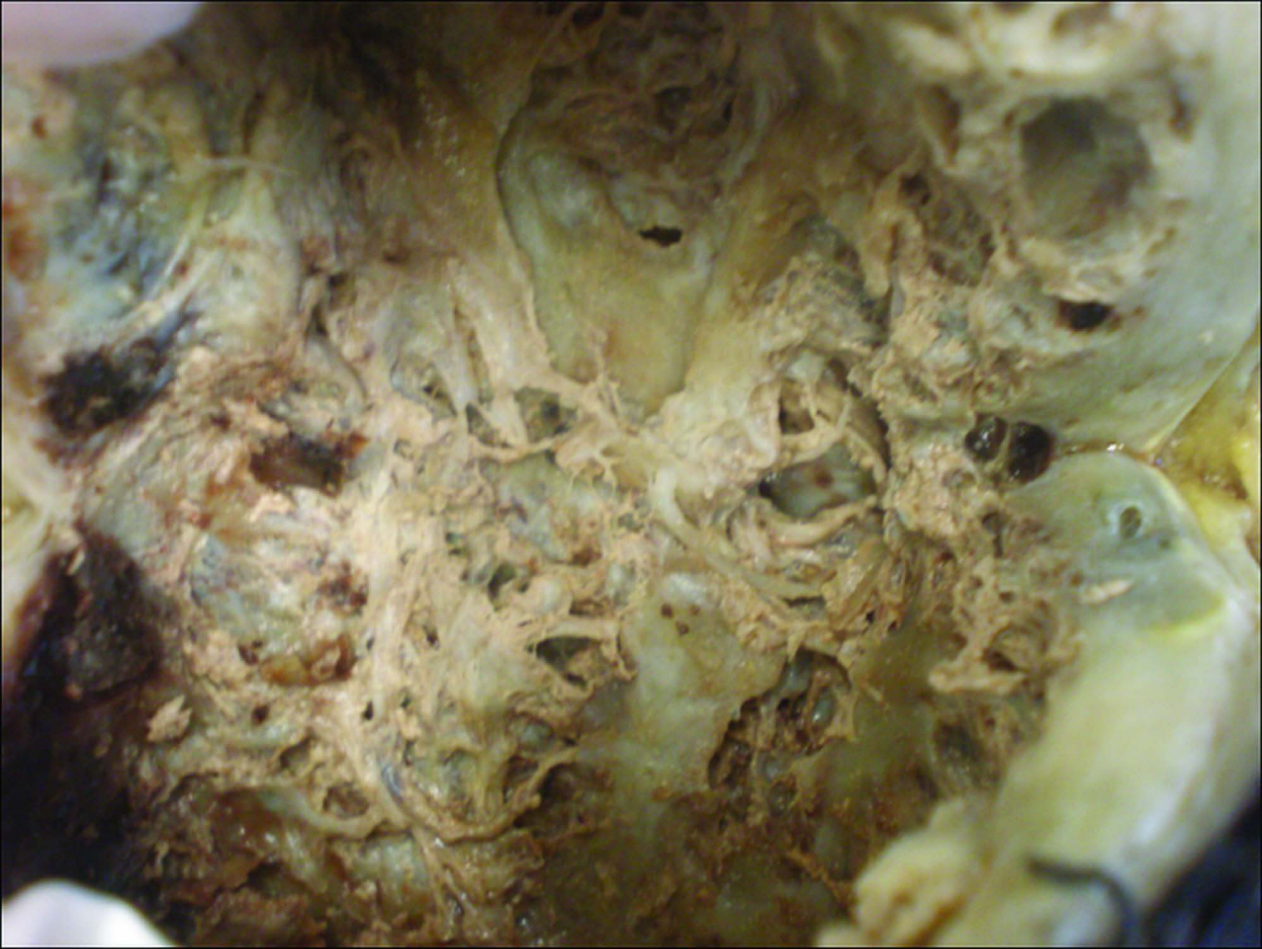
**Gross section demonstrated the intermixed fibrous and myxoid components**.

**Figure 4:**
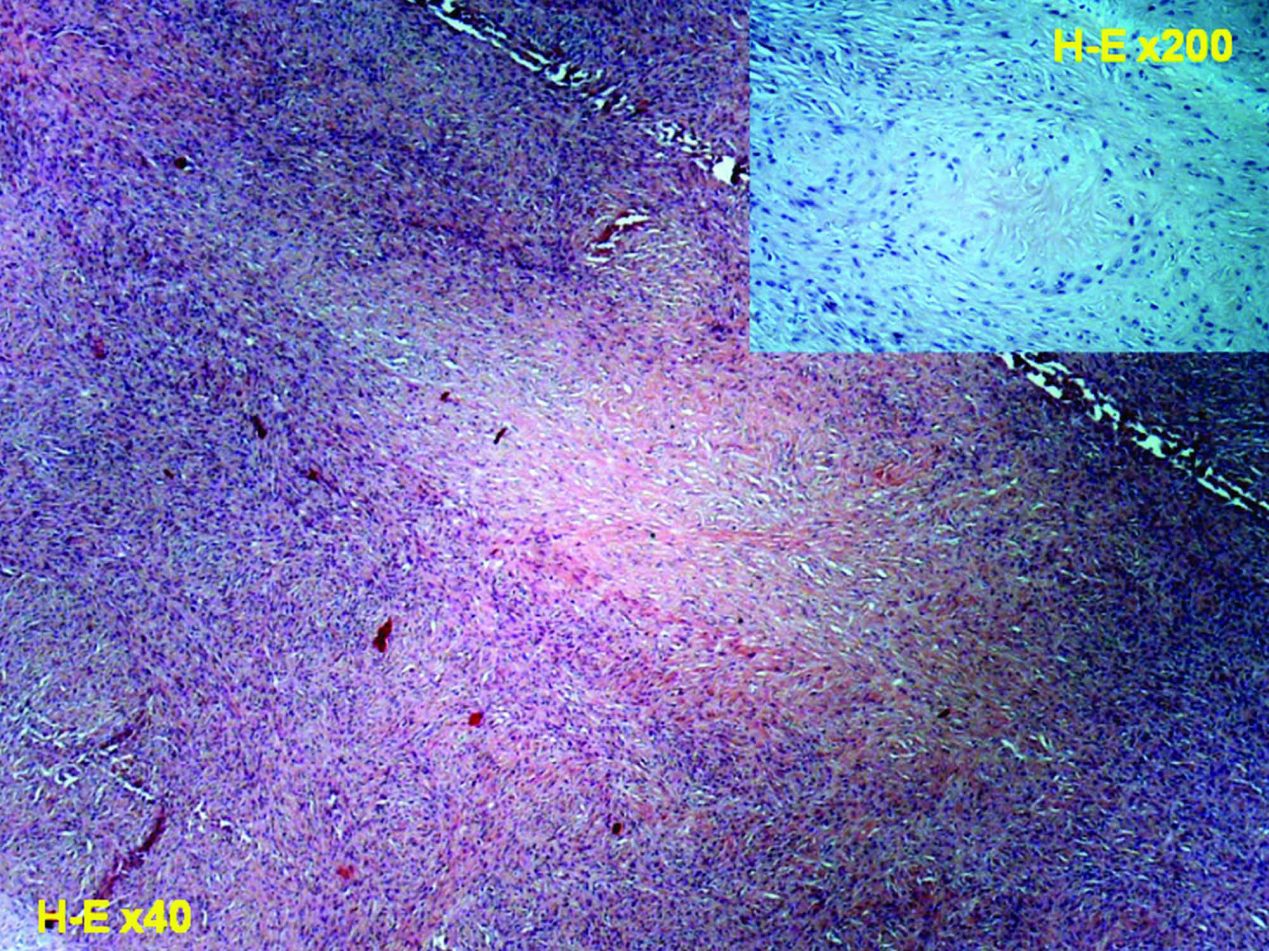
**Giant rosettes with hyallinized central collagen surrounded by plump to oval cells**.

The surgical specimen was sent to the Pathology laboratory. Macroscopically, the tumour measured 11 × 10 × 9 cm and was partially covered by regional muscle fibers (Fig. 2). The surface of the tumor was smooth and glistering with a light tan color. On cut sections a central cystic area filled with partially hemorrhagic, and partially seromucinous fluid was revealed (Fig. 3). The cut surface of the peripheral solid area showed whitish yellow tissue with focal gelatinous appearance of elastic consistency. The microscopical evaluation of the mass revealed a tumor with features similar to those detected in the preceded biopsy. Additionally, the lesion was well-circumscribed, characterized by fibrous areas with increased cellularity and eosinophilic collagen fibers alternating with less cellular, paler myxoid areas. Characteristically giant rosettes were noted in the periphery (Fig. 4). The central cystic area corresponded to a necrotic area. Immunohistochemistry, the neoplastic cells were consistently positive for vimentin only and negative for a variety of antibodies. Occasional cells were positive for SMA. Based on the microscopic features, the diagnosis of LGFMS was made.

Following diagnosis, a CT scan of the chest was performed revealing no metastasis to the lung. Postoperatively, the patient received radiotherapy consisting of a total of 60 cGy (2 cGy daily for 30 days), as well as. One year later the patient is well with no evidence of disease.

## Discussion

Low grade fibromyxoid sarcoma (LGFMS) is a distinctive variant of fibrosarcoma [9]. It is characterized by an admixture of collagenized hypocellular zones and more cellular myxoid nodules. Tumour cells are usually small, with scanty eosinophilic cytoplasm, round to ovoid nuclei and absent nucleoli. Although focal cytologically atypical areas of high cellularity, increased mitotic activity, nuclear hyperchromatism, and necrosis may be found in approximately 10% of the cases, tumour cells are usually characterized by absent to sparse mitotic figures, nuclear anaplasia or necrosis [2]. Immunohistochemical staining is positive for vimentin only and negative with a variety of antibodies, such as desmin, keratin, S100 protein, epithelial membrane antigen, CD34, and CD31. Muscle specific actin is positive in the wall of small vessels within the tumor and strongly positive in the peripheral fibrous layer.

Ten years after the first description of LGFMS by Evans [1], a similar pathologic entity, the hyalinizing spindle cell tumor with giant rosettes (HSTGR) was reported [10]. These tumors are characterized by a proliferation of bland spindle cells with fibromyxoid areas that resemble histologically LGFMS. Giant rosettes which are found in HSTGR is a distinctive pattern that is defined by the presence of hyalinized acellular islands surrounded by oval and spindle cells. In a large series of 77 cases of LGFMS and HSTGR, Folpe et al. [9] supported that these tumors represent the same neoplastic process and are of the spectrum of low-grade sarcomas. According to the same authors, several cases of LGFMS presented the pattern of miniature rosettes that had been overlooked at the time of the initial diagnoses. On the other hand, HSTGR is typically characterized by large areas that are histologically identical to LGFMS. Based on these finding the authors recommended that both entities should be referred to as "fibrosarcoma, low-grade fibromyxoid type" noting either the presence or absence of rosettes. More recently, the view that these two entities share the same pathologic mechanism was strongly supported by several investigators. More specifically, the presence of a balanced t(7;16)(q34;p11) translocation and a fusion between the FUS and CREB3L2 genes in both LGFMS and HSTGR were confirmed by multiple studies [11-16]. This translocation seems to be specific for the diagnosis of these tumors and particularly useful in cases of limited material for examination or in cases that the typical histopathologic features are not present. Furthermore, the FUS/CREB3L1 fusion transcripts of LGFMS can be reliably detected in paraffin-embedded tissues using RT-PCR [17].

Differential diagnosis of LGFMS includes lesions showing spindle cell proliferations with myxoid pattern with or without fibrous component [4]. The entities with predominantly myxoid pattern without significant fibrous component include myxomas, low-grade myxofibrosarcoma, angiomyxomas, myxoid liposarcoma, and myxoid neurofibroma. Tumors with mixed myxoid and fibrous morphologies include neurofibroma, fibromatosis, perineurioma, malignant peripheral sheath tumor, and fibrous histiocytoma. Additional entities that should be encountered are desmoid tumor, desmoplastic fibrosarcoma, and low grade dedifferentiated liposarcoma.

The diagnosis of LGFMS or HSTGR is usually not difficult if the tumor has been removed completely and all the characteristic morphologic and immunophenotypic features described above are present. This is not usually feasible when the material derives from fine needle aspiration or needle core biopsy [4]. In such cases, a wider biopsy, as in our case, or even an excisional biopsy should be performed. If a myxoid pattern is present and the diagnosis still remains unclear, cytogenetics should be requested in order to exclude the rare case of LGFMS [4].

The clinical presentation is usually long-standing and is mainly related to the anatomic location of the mass. LGFMS usually presents as a painless soft-tissue mass with a pre-biopsy duration of over 5 years in 15% of patients [2]. In rare occasions acute presentations of the disease may occur, such as acute respiratory distress and chest pain in case of chest wall LGFMS or seizure activity in a patient with intracranial LGFMS [3,18].

Although imaging findings of LGFMS are nonspecific, certain CT and MRI findings have been described [3,19-21]. On noncontrast CT images the fibrous component of these tumors has been described as isodense to muscle tissue and the myxoid component as hypodense. On MR imaging, the fibrous component is characterized as hypointense on T1- and T2-weighted MR images, and slightly enhancing on T1-weighted MR images after gadolinium administration. On the other hand, the myxoid component has been described as hypointense on T1-weighted MR images and hyperintense on T2-weighted MR images, and vividly enhancing on T1-weighted MR images after gadolinium administration. Calcifications may also be found within the tumor [21].

Evans [6] and Goodlad et al. [8] suggested that LGFMS were paradoxically aggressive tumors. In these retrospective early series, local recurrence was noted in 68%, metastasis in 41%, and death from disease in 18% [2]. Almost all these patients in these original studies were initially diagnosed with, and treated for a benign lesion. It is obvious that patient selection has influenced the reported rate of recurrence and metastases, as many of those cases were selected on the base of unexplained metastases. In a more recent and larger series local recurrence, metastasis, and death from disease was observed in 54%, 6%, and 2% of the patients, respectively [9]. In the same study it has been shown that the presence of focal areas of high cellularity, nuclear enlargement, increased mitotic activity, and necrosis are not of prognostic significance for recurrence or metastasis. Given the potential of LGFMS for late metastasis, sometimes 45 years after initial diagnosis, the median follow-up of only 24 months in the previous study should be considered too short in order to ascertain safe conclusions [4].

Once the diagnosis of LGFMS or HSTGR is made, a full oncological assessment should follow. This should include a CT scan of the chest, since metastasis to the lung is the most common scenario. Because of the high risk of late metastasis, clinical follow-up and chest imaging should be performed for an extended period of time. However, it is still unclear how regularly imaging of the chest should be repeated [4].

## Conclusions

The case report presented herein, enriches the literature with information on imaging diagnosis and surgical treatment of this rare tumor. As there is no dedicated protocol regarding follow-up examinations and in order to early diagnose possible metastasis it is important to inform the patients about the longstanding metastatic potential of the disease.

## Consent

Written informed consent was obtained from the patient for publication of this case report and any accompanying images. A copy of the written consent is available for review by the Editor-in-Chief of this journal.

## Competing interests

There are no competing interests; this is a basic academic research initiative.

## Authors' contributions

All authors contributed equally to this work. MGL, CA and MD participated in the design of the study and drafted the manuscript. IDG and AG participated in the design of the study. TAX and AB conceived of the study, and participated in its design and coordination and helped to draft the manuscript

MGL has had the main responsibility for the study and manuscript preparation. All authors read and approved the final manuscript.
